# Examining the translational success of an initiative to accelerate the assessment of chest pain for patients in an Australian emergency department: a pre-post study

**DOI:** 10.1186/s12913-020-05296-1

**Published:** 2020-05-13

**Authors:** Jaimi H. Greenslade, Ariel Ho, Tracey Hawkins, William Parsonage, Julia Crilly, Louise Cullen

**Affiliations:** 1 Australian Centre for Health Services Innovation (AusHSI) and School of Public Health and Social Work, Faculty of Health, Queensland University of Technology, 60 Musk Avenue, Kelvin Grove, QLD 4059 Australia; 2 Emergency and Trauma Centre, Royal Brisbane and Women’s Hospital, Butterfield Street, Herston, QLD 4029 Australia; 3 School of Medicine, Faculty of Health and Behavioural Sciences, The University of Queensland, 288 Herston Road, Herston, QLD 4006 Australia; 4 Department of Cardiology, Royal Brisbane and Women’s Hospital, Butterfield Street, Herston, QLD 4029 Australia; 5 Department of Emergency Medicine, Gold Coast Health, Southport, QLD 4215 Australia; 6 Menzies Health Institute Queensland, Gold Coast campus, Griffith University, Mount Gravatt, QLD 4222 Australia

**Keywords:** Chest pain, Emergency department, Risk stratification, Translation, Sustainability, Evidence

## Abstract

**Background:**

The Improved assessment of chest pain trial (IMPACT) protocol is an accelerated strategy for the risk stratification and management of patients presenting to the emergency department (ED) with chest pain. This study sought to describe the adoption, sustainability and health services implications of implementing the IMPACT protocol.

**Methods:**

This was a study of adult patients in a large Australian tertiary hospital who had serial troponin testing commenced within the ED. Data from two periods were utilized; the pre-implementation period (8th April 2012 to 5th April 2014) and the post-implementation period (6th April 2014 to 2nd April 2016). The primary outcome was the proportion of patients undergoing accelerated care. Secondary endpoints were ED assessment time, hospital length of stay, and costs. Data were compared in the pre- and post-implementation periods.

**Results:**

The proportion of patients receiving accelerated care increased from 3% in the pre- to 34% in the post-intervention period. This increase occurred rapidly after implementation of IMPACT and was sustained over a 2-year period. For patients with troponin concentrations <99th percentile, the mean ED assessment time reduced from 12.3 h in the pre- to 10.1 h in the post-implementation period. Mean hospital length of stay was similar in the pre- and post-implementation periods (82.4 and 80.9 h). The average cost of chest pain assessment reduced from $3520 pre implementation to $3204 post implementation; a $316 reduction per patient.

**Conclusions:**

The IMPACT protocol was rapidly adopted and utilised after implementation into standard care. The initial increase in the proportion of patients undergoing accelerated assessment, followed by a plateau towards the end of the study period indicate adoption and sustainability of the IMPACT protocol over a two-year period. Modest reductions in length of stay and cost were seen after implementation. Given the large number of patients investigated for chest pain, such reductions may have substantial impact on the overall healthcare system.

## Background

Over 7 million patients present to United States Emergency Departments (ED) each year for investigation of acute coronary syndrome, [1] yet less than 15% will ultimately have the diagnosis confirmed [2]. The traditional assessment of patients with potential acute coronary syndrome incorporates clinical history, electrocardiograms, and serial cardiac troponin testing over 3 to 12 h when using a sensitive troponin assay [3, 4]. Patients with negative results after this initial assessment are then referred for functional or anatomical testing for ischemia [4]. This process takes an average of 34 h per patient [5] and is incongruent with the need to rapidly and safely assess patients in overcrowded EDs.

A number of approaches have been proposed to accelerate patient assessment. One such approach was outlined in the IMProved Assessment of Chest pain Trial (IMPACT) [6]. The IMPACT protocol is a complete strategy for the risk stratification and management of patients with chest pain. Patients are stratified based on clinical history, presentation troponin, and electrocardiogram findings. Low-risk patients undergo 2-h troponin testing before discharge, while intermediate risk patients undergo 2-h troponin testing followed by early functional testing with an exercise stress test where appropriate. High-risk patients undergo traditional guideline-based care, including 6-h troponins and referral for cardiology review. An intervention trial found that 76% of patients were low- to intermediate-risk and, thus, eligible for accelerated assessment [6]. This protocol was implemented as guideline-based care in one institution in 2014 and has since been implemented at nine additional sites [7].

Translation of research into clinical practice is difficult. Findings from tightly controlled clinical trials that only recruit a subset of patients may not be as effective once implemented within standard care. Further, incorporating protocols into guidelines does not guarantee acceptance by physicians; clinicians may not be aware of guidelines, may lack the confidence to act on them, or may not have the knowledge to apply them correctly [8]. To date, there has been limited research examining how well accelerated chest pain assessment strategies are translated into standard care (exceptions include [9, 10]).

Within this study, we sought to describe the adoption, sustainability and health services implications of the IMPACT protocol at one study site. Specifically, the aims were to compare the proportion of patients undergoing accelerated care, length of stay, and direct costs in the periods before and after implementation of the IMPACT protocol as standard care.

## Methods

### Study design and setting

This was a retrospective cohort study using routinely collected hospital administrative data from adult patients presenting to the ED of a single large tertiary hospital in Brisbane, Australia. This is an adult ED providing a complete range of specialist treatment with an annual census of 80,000 patients. The hospital has almost 1000 beds and employs 6000 multidisciplinary staff. Data from two periods were utilized. These included the two-years before IMPACT was implemented (8th April 2012 to 5th April 2014) and 2 years after IMPACT was implemented (6th April 2014 to 2rd April 2016).

### Selection of participants

Patients were included if they had one or more troponin tests ordered within the ED. Troponin testing was used to define the cohort as measurement of troponin is a required component of the assessment and diagnosis of acute coronary syndrome. Serial troponin ordering is not recommended for any other purpose within the study site. Patients were excluded from the primary analyses if 1) they had only one troponin test ordered within 24 h of ED presentation and this troponin was below the 99th percentile. Such patients did not represent those investigated for acute coronary syndrome according to the hospital’s chest pain assessment process. Further, a previous unpublished audit of patients with single troponin tests at our institution revealed that only 4% of such patients were thought to have possible cardiac chest pain. Patients were also excluded if 2) they were transferred to another facility within the first 24 h after ED presentation or 3) were deceased within the first 24 h after ED presentation, as the intended management of such patients is unknown. Finally, the IMPACT trial was being undertaken during the pre-implementation period. Specifically, 786 patients were enrolled in the clinical trial and were managed by a designated research nurse according to the IMPACT protocol. Such patients were excluded from this study as their assessment and movement through the ED did not reflect usual care. Additional file 1 provides the characteristics of the pre-implementation cohort with and without IMPACT patients.

### Intervention

In the pre-implementation period, the 2006 and 2011 National Heart Foundation/Cardiac Society of Australia and New Zealand (NHF/CSANZ) guidelines [11, 12] were in use at the study site. Such guidelines utilize serial troponin testing as part of stratifying patients into low-, intermediate- and high-risk categories. Serial troponin testing is recommended over a 6- to 8-h period when using a sensitive (contemporary) cTn assay. The guidelines recommend that patients with low risk clinical features, normal serial troponin results and normal electrocardiograms can be discharged from ED. This is less than 2% of patients [13]. Patients with intermediate-risk clinical features, normal serial troponin results and normal electrocardiograms are sent for further objective testing, the most commonly used being an exercise stress test. This is approximately 65% of all patients [13]. Patients with high-risk clinical features, troponin concentrations >99th percentile, or abnormal electrocardiograms require admission to hospital and intensive management, often including early invasive investigations.

In the post-implementation period, the IMPACT protocol was standard care 24 h per day, 7 days per week. The IMPACT protocol stratifies patients into low-, intermediate- and high-risk groups using demographic and clinical features (Additional file 1). These features differ from that outlined in the NHF/CSANZ guidelines. All patients undergo a blood test for assessment of troponin concentrations at presentation (zero hours). Low and intermediate risk patients then have a second test at 2 h. Low risk patients are discharged after normal troponin and electrocardiogram results. In the IMPACT trial, approximately 18% of patients were deemed low risk [6]. Intermediate-risk patients with normal troponin and electrocardiogram results receive an in-patient exercise stress test. This was approximately 58% of patients [6]. The assessment and treatment for high-risk patients were unchanged under the IMPACT protocol; such patients were treated as per the NHF/CSANZ guidelines [11, 12].

Based on the i-PARIHS framework [12], implementation of IMPACT occurred after a local experienced study team conducted research showing that existing processes of care were inefficient and costly. During this period, key stakeholders were engaged and became invested clinical champions for the development of IMPACT and its implementation. The research team produced evidence that the IMPACT intervention was safe and provided health services benefits in the local context. Results were widely disseminated to staff within the department through publication and face-to-face education. All ED staff were informed of IMPACT and were empowered to use the pathway, regardless of their position. The intervention did not require additional staff resources as the facilitators were established clinicians involved from inception with an exceptional knowledge of the intervention. Clear flow charts and posters using a traffic light colour system were developed and placed in strategic positions within the department to support ongoing implementation.

### Measurements

The study used data from three administrative databases used by the hospital. Data collected from the ED database included arrival date and time, ED discharge date and time, sex, age, and disposition (home, admitted, transferred, deceased). Troponin concentrations and time of troponin ordering were obtained from the hospital’s pathology database. Hospital discharge time was obtained from the hospital’s admissions database. These data were linked by the ED data manager using deterministic linkage based on the hospital’s unique identifier.

### Outcomes

The primary outcome in this study was the proportion of patients undergoing accelerated chest pain assessment within the ED. This is a measure of the adoption of the protocol. As this study used administrative data, no data were available on the actual risk or management of patients within the ED. However, the IMPACT protocol enabled accelerated assessment of low- to intermediate- risk patients through the use of 0- and 2-h troponin testing rather than 0- and 6-h testing. As such, we defined accelerated chest pain assessment as having a non-elevated troponin test on presentation (0 h), a repeat test at 2 h, and no 6-h test. As troponins may not be performed exactly within the recommended time period and errors in the time entered on the database are also possible, a presentation troponin was considered to be any troponin taken within 1.5 h of presentation and a two-hour troponin was any troponin taken within 4 h of presentation. Patients were also considered to have accelerated assessment if they had two troponins taken less than 3 h apart within the first 4.5 h after presentation.

Secondary endpoints were used to measure health services impacts, including ED assessment time, hospital length of stay, and costs. ED assessment time included time spent in the ED and/or short stay unit. This was chosen as it represents the emergency assessment period for chest pain patients presenting with chest pain at the study site. The IMPACT protocol did not seek to alter care for high-risk patients. Thus, length of stay was reported separately for the entire cohort and for the cohort excluding high-risk patients. With the actual risk stratification of patients being unknown, patients were deemed high-risk if they had a troponin concentration > 99th percentile. Direct hospital costs were calculated using the cost prediction model detailed by Jülicher et al. [5]. This approach uses a regression equation to estimate the costs associated with chest pain assessment for each patient. The equation incorporates ED length of stay, hospital admission, hospital length of stay, and type of cardiac testing undertaken. This equation estimated actual hospital costs with accuracy in previous Australian research [5]. The equation was derived using 2011 costs. Thus, after calculation, the costs were adjusted for inflation to 2016 AUD using the rate provided by the Reserve Bank of Australia [14].

### Analysis

Data were analysed using Stata 14 (StataCorp, 2015, College Station, TX). Data included 104 weeks (2 years) during the IMPACT intervention period, and 104 weeks (2 years) during the post-implementation period. Demographic and presentation data were provided to characterize the pre-and post-implementation cohorts. Descriptive statistics for the primary outcome (number of patients undergoing accelerated assessment) and secondary outcomes (ED Length of stay, hospital length of stay and cost) were also reported by study period. Differences between the study periods with 95% confidence intervals (CI) were computed. Clustered robust 95% CIs were reported to account for patients having multiple presentations during the study period. The secondary outcomes focussing on length of stay (ED assessment period and hospital length of stay) were right skewed. Accordingly, means were reported and the 95% CIs of the difference between means were computed using a gamma distribution. Medians were also reported for such outcomes to allow comparison with previous research.

To provide details on the uptake and sustainability of IMPACT across time, a generalized linear model was fit regressing accelerated care on time (in weeks) and study period (to allow for a discontinuity at the start of the post-implementation period). The purpose of this analysis was to describe the data across time, and we utilised restricted cubic splines to allow for non-linearity in the odds of accelerated care across time (Additional file 1 provides details regarding the placement of these splines). A binomial error distribution and a logit link function was specified. Robust clustered standard errors were calculated due to mild non-independence within the data. Analyses were also repeated using segmented regression analyses to identify the immediate impact and change in slope associated with the intervention. The results of such analyses mirrored those from the general linear model above and are reported in Additional file 1.

The management of high-risk patients, and particularly those with ST-segment myocardial infarction changed during the pre-implementation period such that these patients were immediately transferred for cardiology review (and cardiac catheterization where appropriate) rather than being processed through the ED. This meant that the proportion of high-risk patients reduced over the study period. To ensure that this difference did not bias the comparisons of the outcome variables across time, the cohort was weighted using iterative post-stratification to ensure that the marginal distributions of the baseline characteristics were similar across the study period. The variables matched were age (ten-year bands), sex, and percentage of patients with an elevated troponin. Both weighted and unweighted data were reported for all comparisons.

The direct hospital cost for each patient was estimated using the cost prediction equation detailed by Jülicher et al [5]. To account for uncertainty in the regression coefficients used to predict costs, we estimated each patient’s cost 10,000 times based on probabilistic sampling of each of the model coefficients. The average of these 10,000 estimates and differences between the averages are reported (See Additional file 1 for further details). Further, when calculating the model, no data were available on cardiac testing. As such, we excluded high-risk patients from the cost analyses. The recommended care for these patients was not altered by implementation of the IMPACT protocol and without data on cardiac testing, the costs for such patients would be inaccurate as they potentially undergo a wide range of costly tests. The remainder of patients were all assumed to undergo the cheapest and most commonly used cardiac test (exercise stress test). Presuming that all patients underwent exercise stress testing is a conservative approach as the IMPACT trial found that IMPACT patients received fewer and less costly tests. The approach taken will, thus, likely underestimate the differences in total costs.

## Results

There were 50,006 troponin tests that could potentially be linked to an ED presentation during the study period. Of those, 46,794 (94%) were successfully linked. For the remaining 6% (*n* = 3212), 3044 were tests taken in an inpatient ward or an outpatient clinic and only 168 (0.3%) were taken in the ED. Of the 46,794 successfully linked tests, 5825 (12%) were excluded as they were from a presentation where the patient did not have any troponin testing performed in the ED; that is, all troponin tests were taken after admission to the ward. This left 40,969 linked tests across 20,316 ED presentations.

**Fig. 1:**
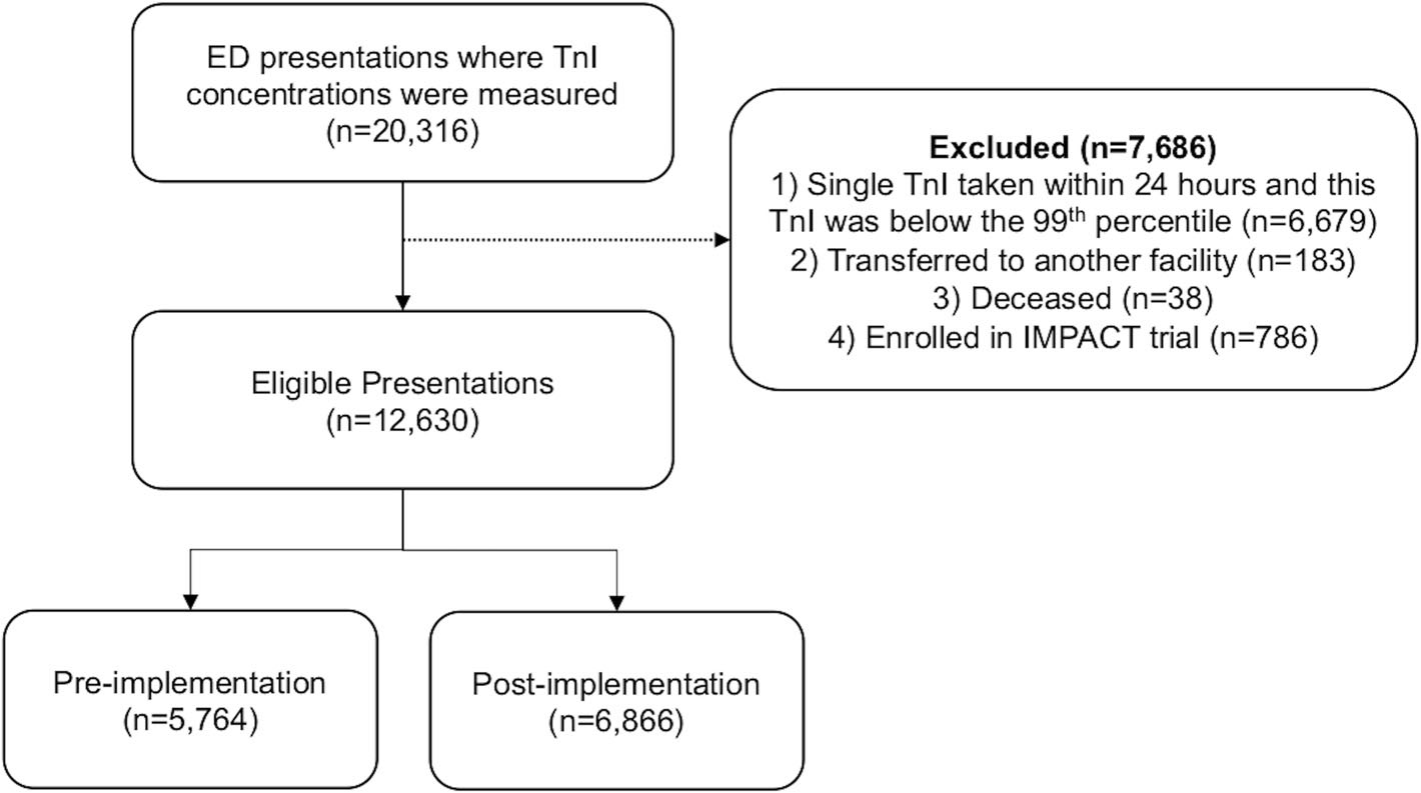
Study flow chart

There were 7686 episodes meeting the exclusion criteria, leaving 12,630 presentations eligible for this study (Fig. 1). These exclusions were for single troponin testing (*n* = 6679), being transferred to another facility (*n* = 183), deceased (*n* = 38) and being enrolled in the IMPACT trial (*n* = 786). Baseline characteristics are shown in Table 1. As identified previously, the changing management of high-risk patients meant that there were a higher proportion of patients with an elevated troponin in the pre- (32.6%) compared to post-implementation (20.6%) periods. This imbalance was removed by weighting the data; the proportions of high-risk patients after weighting were 32.6% (95% CI: 30.8 to 34.4%) in the pre-implementation period and 32.6% (95% CI: 30.8–34.4%) in the post-implementation period.

**Table 1:** Baseline characteristics of the study cohort

Characteristic	Pre-Implementation* (*n* = 5764)	Post-Implementation* (*n* = 6866)	Difference (95% CI)
Mean age (SD)	61.0 (17.2)	58.1 (17.0)	−2.9 (−3.5 to − 2.1)
Male sex	3366 (58.4%)	4034 (58.8%)	0.4% (−1.6 to 2.3)
Mean time to zero-hour troponin (SD)	0.5 (0.3)	0.5 (0.3)	0.0 (−0.0 to 0.0)
Presented after work hours	3325 (57.7%)	3729 (54.3%)	−3.4% (−5.2 to −1.6)
Troponin >99th percentile on zero- or two-hour test	1877 (32.6%)	1412 (20.6%)	−12% (− 13.8 to − 10.2)
Disposition from ED			
Admitted to inpatient ward	3364 (58.4%)	3366 (49.0%)	−9.3% (−11.2 to −7.5)
Admitted to short stay	2005 (34.8%)	2439 (35.5%)	0.7% (−1.0 to 2.5)
Discharged home	371 (6.4%)	1010 (14.7%)	8.3% (7.2 to 9.4)
Left against medical advice	24 (0.4%)	51 (0.7%)	0.3% (0.1 to 0.6)

*CI* confidence interval, *SD* standard deviation, *ED* emergency department

*Data are mean (SD) for continuous variables and n (%) for categorical variables

### Adoption

Accelerated assessment was provided to a higher proportion of patients in the post-implementation group (*n* = 173, [3.0%] pre-implementation and *n* = 2878 [41.9%] post-implementation). After applying weightings, the proportion of accelerated patients was 3.0% in the pre- and 34.4% in the post-implementation period (Table 2).

**Table 2:** Health care utilization. Data are unweighted or weighted by age, sex and troponin

	Unweighted Data			Weighted Data		
	Pre-Implementation* (*n* = 5764)	Post-Implementation* (*n* = 6866)	Difference (95% CI)	Pre-Implementation†	Post-Implementation†	Difference (95% CI)
Proportion of patients accelerated	173 (3.0%)	2878 (41.9%)	39.0% (37.5 to 40.3%)	3.0% (2.6–3.5%)	34.4% (33.1 to 35.8%)	31.4% (30.0 to 32.8%)
**ED assessment period, hours**						
Median (IQR)	9.0 (5.9 to 14.8)	7.4 (4.8 to 12.5)	−1.6 (−1.8 to − 1.4)	9.0 (5.9–14.8)	7.4 (4.8 to 12.1)	−1.6 (− 1.8 to − 1.4)
Mean (95% CI)	11.1 (10.9 to 11.3)	9.7 (9.5–9.9)	−1.4 (− 1.7 to − 1.1)	11.1 (10.9–11.3)	9.5 (9.4 to 9.7)	−1.5 (− 1.8 to − 1.2)
**ED assessment period excluding high risk patients, hours**						
Median (IQR)	10 (7.0 to 17.3)	7.5 (4.9 to 13.5)	−2.5 (−2.8 to − 2.2)	10 (7.0 to 17.3)	7.6 (4.9 to 13.5)	−2.4 (− 2.7 to − 2.1)
Mean (95% CI)	12.3 (12.1 to 12.6)	10.1 (9.9 to 10.3	−2.3 (− 2.6 to − 2.0)	12.3 (12.1 to 12.6)	10.1 (9.9 to 10.3)	−2.3 (− 2.6 to − 2.0)
**Hospital length of stay, hours**						
Median (IQR)	36.7 (16.8–97.3)	24.7 (8.1 to 78.5)	−12.1 (− 14.8 to −9.4)	36.7 (16.8–97.3)	34.4 (10.5 to 99.6)	−2.3 (−6.0 to 1.3)
Mean (95% CI)	82.6 (78.7 to 86.5)	67.5 (64.5 to 70.5)	−15.1 (− 19.8 to − 10.4)	82.4 (78.6 to 86.2)	80.9 (77.1 to 84.7)	−1.5 (− 6.7 to 3.7)
**Hospital length of stay excluding high risk patients, hours**						
Median (IQR)	24.0 (13.7–58.90)	19.5 (6.9 to 51.7)	−4.5 (−5.4 to −3.6)	24.0 (13.6 to 59.5)	20.3 (7.3 to 54.2)	−3.7 (−4.7 to −2.8)
Mean (95% CI)	50.2 (47.7 to 52.9)	47.9 (45.5 to 50.3)	−2.4 (−5.8 to 1.1)	50.4 (47.7 to 52.8)	50.2 (47.7 to 52.7)	−0.2 (−3.7 to 3.3)

*CI* confidence interval, *IQR* Interquartile range, *ED* Emergency Department. * Data are n (%), mean (95% CI), and median (IQR) † Data are n (95% CI), mean (95% CI) or median (IQR)

### Health services impacts

Weighted and unweighted data on healthcare utilization for the entire cohort are provided in Table 2. The weighted mean ED assessment period (ED and short stay unit length of stay) for all patients was reduced from 11.1 in the pre- to 9.5 h in the post-implementation period. After exclusion of high-risk patients, the mean assessment period reduced by 2 h from 12.3 h in the pre- to 10.1 h in the post-implementation period. Unadjusted mean hospital length of stay was shorter in the post- compared to pre-implementation period, but these differences did not emerge after weighting the data.

The expected costs per patient from 10,000 simulated samples were $3520 (95% CI: $3515–$3525) in the pre-implementation period and $3204 (95% CI: $3200–$3207) in the post-implementation period. This equated to an average saving of $316 (95% CI: $310–322) per patient.

The primary and secondary analyses were repeated using the cohort of patients that included those with single troponin tests. These analyses were performed to identify whether the removal of the large cohort of patients with single troponin tests altered the study results. Within these analyses, all patients with a single troponin test only were considered to have undergone accelerated assessment. As shown in Additional file 1, inclusion of those with single troponin testing did not change the study conclusions. There remained an increase in the proportion of patients undergoing accelerated testing and a modest decrease in the ED assessment time after implementation of the protocol.

### Sustainability

**Fig. 2:**
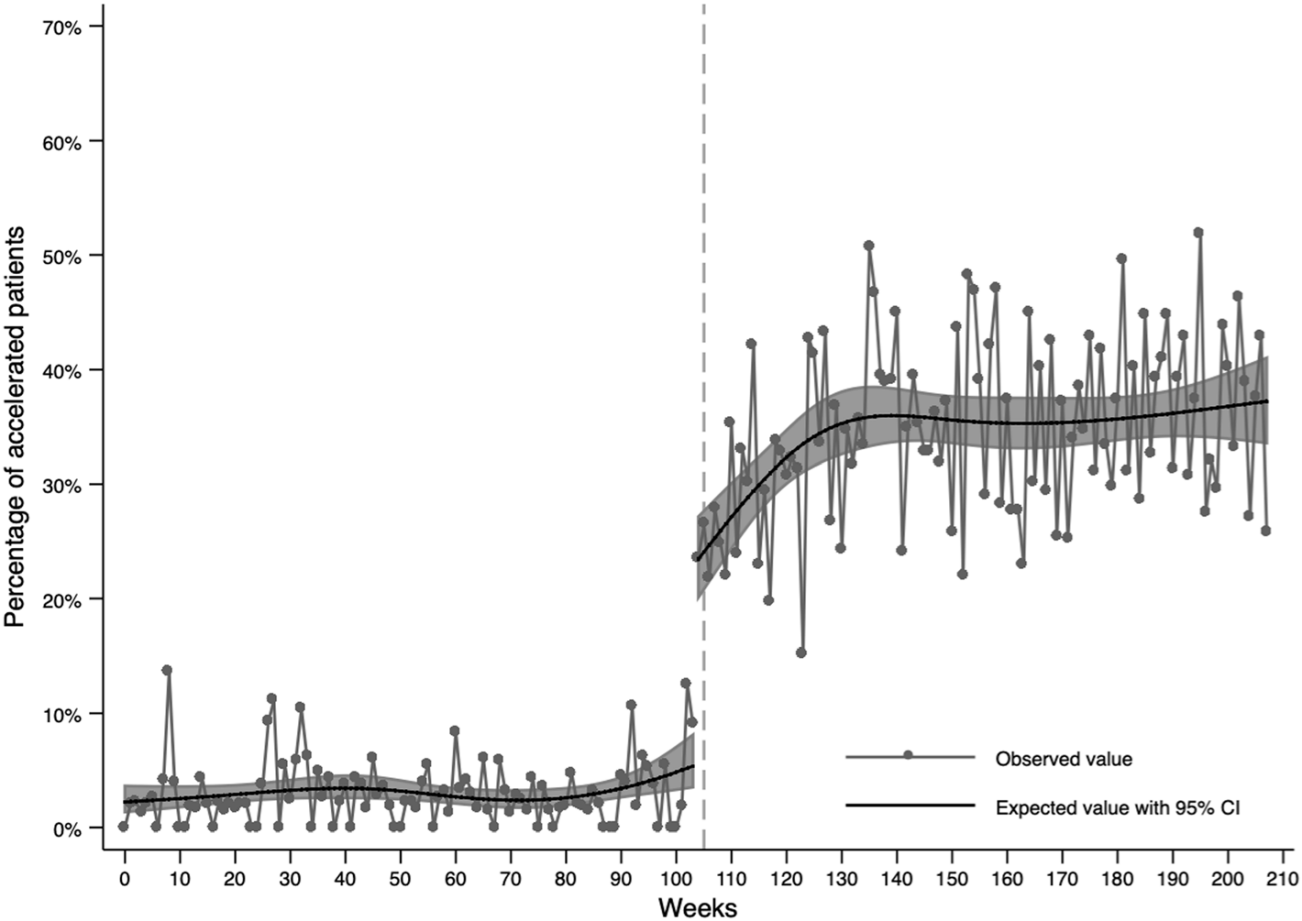
Percentage of patients accelerated by time. The grey dashed line represents the start of the IMPACT implementation period. Data have been weighted by age, sex and elevated troponin to ensure balance in the marginal distributions for these variables across the study period

Figure 2 provides data on the weighted proportion of accelerated patients across the four-year study period (Additional file 1 provides a comparison of weighted and unweighted data). The proportion of accelerated patients was stable across the pre-implementation period. The odds of being accelerated increased 5.2 times (95% CI: 3.0 to 8.9) immediately upon implementation of IMPACT as standard care. Accelerated assessment continued to increase rapidly over the first 6 months after implementation, then plateaued for the remainder of the study period. The weighted proportion of accelerated patients plateaued to an average of 37.2% (95% CI: 33.6 to 41.0%) of the total cohort by the end of the study period.

## Discussion

This study provides data on the adoption, sustainability and health services implications of implementing the IMPACT protocol into standard care. We found that the uptake of the protocol increased in the first year after implementation and was then sustained for a further year. The ED assessment period and estimated direct hospital costs decreased after implementation of IMPACT.

The number of patients undergoing accelerated assessment increased over time following implementation of the protocol. Two years after implementation, around 40% of patients presenting to ED and investigated for chest pain underwent accelerated assessment. This indicates that while the protocol took time to be fully adopted, the use of this pathway has since been sustained. Despite this, the proportion of patients deemed suitable for accelerated assessment was lower than the proportion of patients undergoing only 0- and 2-h troponin testing in the original IMPACT study (66% [unpublished data]). One potential explanation for this difference is that strict inclusion and exclusion criteria where patients enrolled in intervention studies may be less unwell than the general population [15, 16]. This would mean that there were more low- to intermediate-risk patients in the IMPACT cohort compared to the general cohort of ED patients. Indeed, the proportion of patients with zero- or two-hour troponin concentrations >99th percentile (the criteria for high risk) was 9% in the IMPACT study (unpublished data) but 26% in the current study. Additional explanations for the relatively lower number of accelerated patients may be clinician non-adherence to the protocol or the inability to conduct rapid troponin testing due to departmental workload.

This study found modest reductions in length of stay after implementing IMPACT. The ED assessment period was shorter, particularly after the exclusion of high-risk patients. Median (but not mean) hospital length of stay also reduced for low- to medium-risk patients after the implementation of IMPACT. The median differences in hospital length of stay were in line with the limited previous research on the implementation of chest pain pathways into clinical care. For example, implementation of the HEART pathway was associated with a 2-h decrease in hospital length of stay [10]. Implementation of the ADAPT protocol in 16 Australian hospitals was associated with a 10-h reduction in length of stay but the data from individual hospitals were varied, with 6 of the hospitals reporting modest reductions (less than 3 h) [9]. Variability in admission practices and inpatient procedures across different wards and hospitals may make it difficult to achieve large reductions in length of stay following an ED intervention. Despite this, the ED length of stay was reduced meaning that patients were either discharged or admitted earlier, thereby potentially reducing overcrowding, and associated adverse effects, within the ED.

The finding that mean hospital length of stay did not differ after implementation of IMPACT is likely due to the extreme variability in length of stay across patients, in combination with the administrative nature of the data. No data were available on the risk stratification assigned to patients and so all patients with cTn values below the 99th percentile were considered in the comparisons. Patients with high-risk features aside from elevated troponin concentrations were included and those with long length of stays may have obscured mean differences in the pre- and post-implementation periods.

The costs for the assessment of chest pain within the hospital were lower after implementation of IMPACT. The reduction of approximately $300 per patient for the 2500 patients presenting per year with a non-elevated troponin would equate to a yearly cost saving of $750,000 at the study site alone. This cost saving is similar to that identified in research modelling the impact of moving from a 6-h to a 2-h troponin test for low- to intermediate-risk patients [5]. However, the saving was lower than the estimated $1300 per patient reduction found in the original IMPACT trial [17]. As noted previously, the predicted cost savings are likely to be an under-estimate as costs were estimated presuming that all patients underwent exercise stress testing. The IMPACT protocol allows for low-risk patients to be discharged without exercise stress testing and was shown to reduce more costly testing (such as myocardial perfusion scans).

### Limitations

The study sought to provide an evaluation of the real-world impact of implementing an accelerated chest pain protocol. As such, it utilized administrative data from all patients undergoing troponin testing. However, the limited nature of administrative data means that no data were available on the actual risk stratification assigned to patients. Accelerated assessment was presumed based on the timing of troponin testing and high troponin concentrations were used as an indicator of high-risk. As such, it is unclear how many patients were truly low- to intermediate-risk and could potentially be accelerated according to the IMPACT protocol. This limitation may mean that the results of this study are an under- or overestimate of the true effect of implementing the IMPACT protocol. When using retrospective data, there is the potential that the intervention influences the data collection, both in terms of volume of data and the definitions of data. This can create a bias whereby changes in outcome variables after the intervention are due to heterogeneity of data collection rather than the intervention. However, in the current study, the endpoints used are routinely collected for all patients and are recorded in a standardized manner. Accordingly, we believe the potential bias is low. In the time since completion of data collection for this study, a number of additional chest pain assessment strategies have been assessed. These include the use of risk scores (e.g., HEART, EDACS and MACS) and troponin only algorithms using high sensitivity troponin assays. However, few reports on the implementation of these strategies exist and IMPACT retains several advantages. First, it can be used with any troponin assay and can incorporate high sensitivity troponin algorithms. Second, it is an overall model for chest pain assessment that includes both risk stratification and recommendations for assessment. Third, it identifies a broad range of cardiac outcomes including acute myocardial infarction, acute coronary syndrome and other major cardiac events. This study reflects the outcomes of adoption of research findings at a single centre, engaged in the original research. The enthusiasm for change may have been influenced by the local experience and this may result in an overestimate of the benefits of using this protocol. The cost prediction model utilized in this study has only been used in one previous study. While the estimated mean costs in the current study were in line with previous literature [5, 17], it is unknown whether this model accurately reflects true costs. Again, it is unknown whether this would reduce or increase the estimate of the treatment effect. This study did not include a true control group (e.g. data from a hospital that did not have the intervention implemented). As such, it is unclear whether the changes identified were related to the implementation of IMPACT, or whether other factors contributed to the change. The finding that the outcomes changed immediately after implementation provide some evidence that the change occurred as a result of IMPACT, but causality cannot be assumed. We excluded a sample of patients from the pre-implementation period who were enrolled in the IMPACT trial. While this did not change the demographics of the pre-intervention sample (Additional file 1), the exclusion of such patients may have had an unknown influence on the results. The goal of this study was to track the health services implications of implementing the IMPACT protocol. In this regard, our inability to include data on functional or anatomical testing after assessment is a limitation. Patients were assumed to have undergone accelerated assessment if they had two troponin tests up to 4.5 h after arrival with a maximum time between tests of three hours. As such, if patients had a delayed first test, they were not considered accelerated even if their second test was within 2–3 h of the first. This conservative approach was taken as we could not identify physician intent in this instance; that is, accelerated testing after a delayed first troponin, or intent to perform a 6 to 12-h test. If such patients were instead presumed to be accelerated patients, the proportion of accelerated patients would have been 299/5764 (5.2%) in the pre-implementation period and 3587/6866 (52.2%) in the post-implementation period. Future research could overcome many of these limitations by prospectively collecting data from multiple sites.

## Conclusions

This study demonstrated that the IMPACT protocol was rapidly adopted and utilized after implementation into standard care. The initial increase in proportion of patients undergoing accelerated assessment, followed by a plateau towards the end of the study period indicate adoption and sustainability of the IMPACT protocol over a two-year period. Modest reductions in length of stay and cost were seen. Given the large number of patients investigated for chest pain, such reductions may have substantial impact on the overall healthcare system.

## Supplementary information

**Additional file 1.**

## Data Availability

The authors are not able to make the dataset publicly available due to patient confidentiality policies. All data reported in this study were administrative data collected by Queensland Health. In some circumstances, Queensland Health will provide de-identified data to external parties. JG can be contacted to discuss Queensland Health protocols for applying to obtain access to confidential patient data.

## Abbreviations

ED: Emergency Department
IMPACT: Improved Assessment of Chest Pain Trial
NHF/CSANZ: National Heart Foundation/Cardiac Society of Australia and New Zealand
CI: Confidence interval
