# Supplementary material for: Examining the translational success of an initiative to accelerate the assessment of chest pain for patients in an Australian emergency department: a pre-post study
Source: BMC Health Serv Res. 2020 May 13;20:419. doi: 10.1186/s12913-020-05296-1 (PMC7222586; doi:10.1186/s12913-020-05296-1)

Appendix 1. Baseline characteristics of the pre-implementation cohort before and after removal of the IMPACT trial participants.

Characteristic.	Pre-Implementation all patients (n=6,550)	Pre-Implementation without IMPACT trial (n=5,764)
Mean Age (SD)	59.7 (17.0)	61.0 (17.2)
Male Sex, n (%)	3831 (58.5%)	3366 (58.4%)
Disposition from ED, n (%)		
Admitted to inpatient ward	3,524 (53.8%)	3,364 (58.4%)
Admitted to short stay	2,538 (38.8%)	2005 (34.8%)
Discharged Home	459 (7.0%)	371 (6.4%)
Left against medical advice	27 (0.4)	24 (0.4%)
Inter-hospital transfer (>24h)	2 (0.0%)	0 (0%)
Mean time to zero-hour troponin (SD), hours	0.5 (0.3)	0.5 (0.3)
Presented after standard work hours, n (%)	3,382 (51.6%)	3,325 (57.7%)
Troponin >99th percentile on zero- or two-hour test, n (%)	1939 (29.5%)	1877 (32.6%)

IMPACT=Improved assessment of chest pain trial, SD=standard deviation, ED=emergency department.

Appendix 2. IMPACT protocol

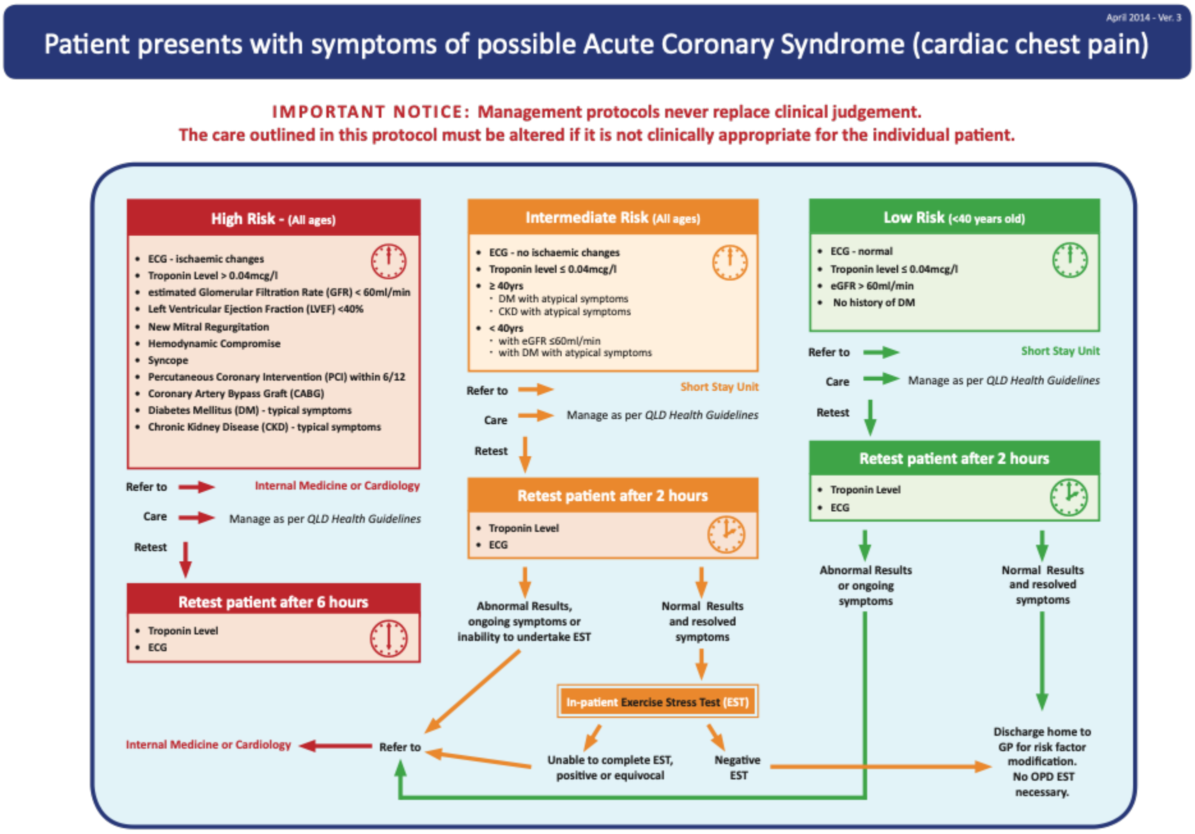

Appendix 3. Details about restricted cubic splines

The purpose of this analysis was to describe the data across time and we did not have a hypothesis about how the odds of accelerated care might change across time (i.e., no relationship, linear, curvilinear). As such, we utilized restricted cubic splines to enable the relationship to be dictated by the data. Splines were set at six-month intervals to capture cooler (March to August) and warmer months (September to February) in Brisbane, Australia. An exception was that a spline knot was set in April 2014 (the start of the post-implementation period) rather than March 2014.

Appendix 4. Segmented regression analysis

We conducted a segmented regression analysis to identify the immediate impact and change in slope associated with the intervention. Three variables were included in these analyses; weeks after study commencement, to assess the trend in accelerated patients in the pre-implementation period; weeks after implementation, to assess a change in trend post implementation of IMPACT; and an indicator variable reflecting whether there was a quantitative change in outcome upon implementation of the IMPACT pathway. The results supported the results reported in the main body of the paper; the proportion of accelerated patients was stable across the pre-implementation period (β=0.0, 95% CI: -0.01 to 0.0). The proportion of patient accelerated increased 27.3% (95% CI: 24.2 to 30.3) immediately upon implementation of IMPACT as standard care. The trend across time changed after implementation of IMPACT (β =0.07, 95% CI: 0.02 to 0.12). This equated to an average of a 0.08% (95% CI: 0.03 to 0.1) increase per week in the proportion of accelerated patients in the post impact period. Partial correlograms and the Cumby-Huizinga test indicated that there was no autocorrelation in the residuals.

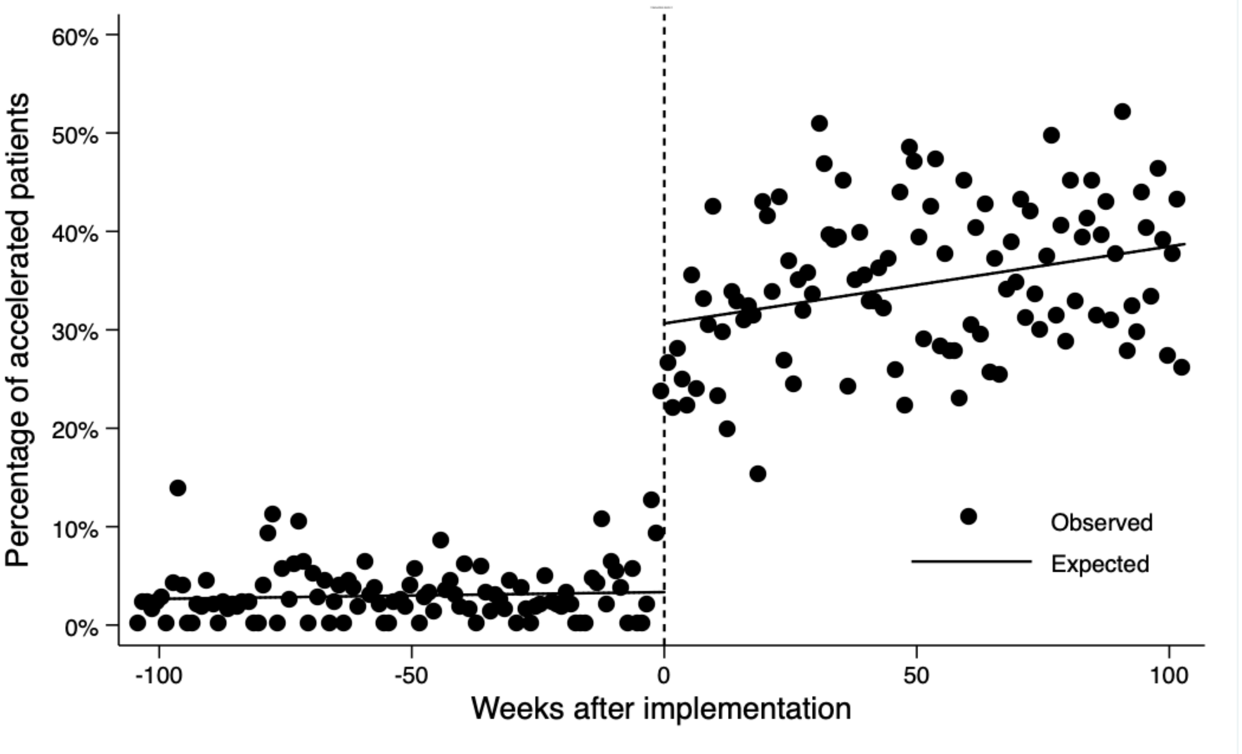

Appendix 5

The direct hospital cost for each patient was estimated using the cost prediction equation detailed by Jülicher et al. (5) In any regression equation, the regression coefficients are estimates and include uncertainty (reported as a standard error). To account for this uncertainty, we estimated each patient's cost 10,000 times based on probabilistic sampling of each of the model coefficients and their standard error. This results in 10,000 estimates of cost per patient, and consequently 10,000 estimates of the average cost for the pre- and post-implementation groups. The average of these 10,000 estimates was calculated for each group (pre- and post-implementation) and the difference between the averages (with 95% confidence intervals) are reported in this paper.

Appendix 6. Health care utilisation for cohort including single troponins. Data are either unweighted or weighted by age, sex and troponin.

	Unweighted Data			Weighted Data		
	Pre * (n=5,764)	Post* (n=6,866)	Difference (95% CI)	Pre†	Post†	Difference (95% CI)
Patients accelerated	3633 (39.4%)	6097 (60.5%)	21.15 (19.5 to 22.6%)	39.4% (38.1 to 40.6)	55.6% (54.4 to 56.9)	16.2% (14.6 to 17.9%)
**ED assessment period, hours**						
Median	7.6	6.6	-1.0	7.6	6.7	-0.9
(IQR)	(4.4 to 2.2)	(4.2 to 11.0)	(-1.1 to -0.80)	(4.4 to 12.2)	(4.2 to 10.9)	(-1.1 to -0.7)
Mean	9.6	8.8	-0.8	9.6	8.8	-0.8
(95% CI)	(9.4 to 9.8)	(8.7 to 9.0)	(-1.0 to -0.6)	(9.4 to 9.7)	(8.6 to 8.9)	(-1.0 to -0.5)
**ED assessment period excluding high risk, hours**						
Median	7.8	6.5	-1.2	7.8	6.6	-1.1
(IQR)	(4.4-13.0)	(4.2-11.2)	(-1.4 to -1.0)	(4.4-13)	(4.2-11.1)	(-1.3 to -1.0)
Mean	9.9	8.9	-0.9	9.9	8.9	-1.0
(95% CI)	(9.7-10.0)	(8.8-9.1)	(-1.2 to -0.7)	(9.7-10.0)	(8.8 -9.1)	(-1.2 to -0.7)
**Hospital Length of stay, hours**						
Median	26.0	22.4	-3.6	26.1	26.1	0.0
(IQR)	(8.5 to 91.2)	(5.7 to 80.0)	(-4.9 to -2.3)	(8.5 to 91.3)	(6.2 to 94.3)	(-1.5 to 1.5)
Mean	74.7	66.9	-7.8	74.8	74.0	-0.8
(95% CI)	(71.5 to 77.9)	(64.3 to 69.5)	(-11.8 to -3.8)	(71.5 to 78.0)	(71.1 to 76.9)	(-5.0 to 3.4)
**Hospital Length of stay excluding high risk, hours**						
Median	20.6	18.4	-2.2	20.6	19.0	-1.6
(IQR)	(7.0 to 64.4)	(5.0 to 63.3)	(-3.2 to -1.1)	(7.0 to 64.4)	(5.1 to 66.4)	(-2.6 to -0.5)
Mean	55.6	54.5	-1.1	55.8	56.1	0.3
(95% CI)	(52.8 to 58.5)	(52.2 to 56.9)	(-4.7 to 2.5)	(52.9 to 58.7)	(53.7 to 58.5)	(-3.4 to 4.0)

CI=confidence interval, IQR=Interquartile range, ED=Emergency Department.

* Data are n (%), mean (95% CI), and median (IQR)

† Data are n (95 % CI), mean (95% CI) or median (IQR)

Appendix 7. Percentage of patients accelerated by time using unweighted and weighted data. The grey dashed vertical line represents the start of the IMPACT implementation period. Data have been weighted by age, sex and elevated troponin to ensure balance in the marginal distributions for these variables across the study period.

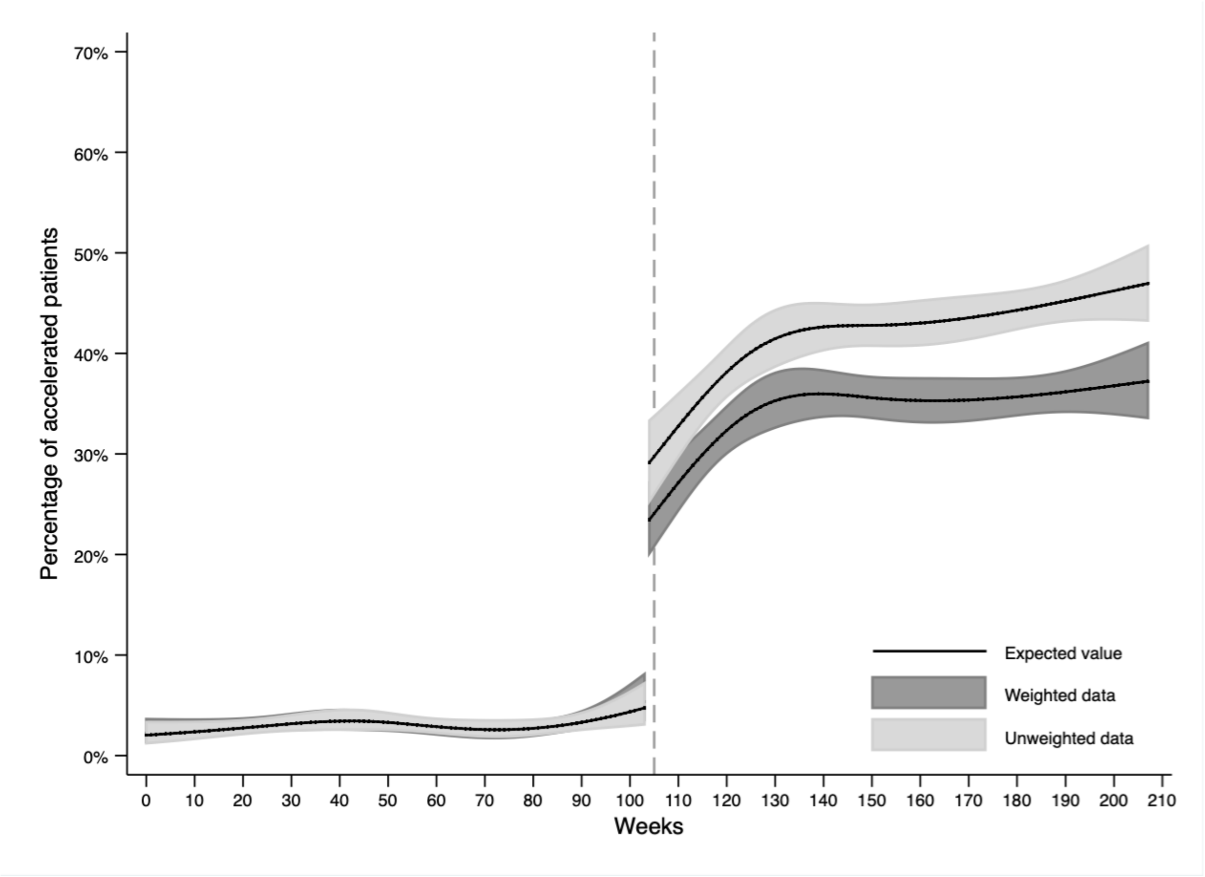

Supplement: Supplementary file 1 — Additional file 1. [file 12913_2020_5296_MOESM1_ESM.pdf]
